# Risk Profiles and Outcomes of Patients Receiving Cardiovascular Implantable Electronic Devices With and Without Antibacterial Envelopes

**DOI:** 10.7759/cureus.24739

**Published:** 2022-05-04

**Authors:** David A Woodard, Grace Kim, Kent R Nilsson

**Affiliations:** 1 Cardiology - Electrophysiology, Piedmont Heart Institute, Athens, USA; 2 Cardiology - Electrophysiology, Augusta University/University of Georgia Medical Partnership, Athens, USA

**Keywords:** cardiac resynchronization therapy, implantable cardioverter-defibrillator, pacemaker, infection, extracellular matrix, envelope, cardiovascular implantable electronic device

## Abstract

Background

The increasing use of cardiac implantable electronic devices (CIEDs) in a growing patient population has led to an even greater increase in CIED infection rates. Antibacterial CIED envelopes are often used as part of an infection risk-reduction strategy. However, best practices for when to use an envelope and which envelope to choose remain to be elucidated.

Methods

In this retrospective study, the records of 455 patients undergoing CIED implantation by a single surgeon were reviewed to identify trends in envelope use and outcomes after implantation through 12 months of follow-up. Of these patients, 165 were managed with a biologic antibacterial CIED envelope (CanGaroo®, Aziyo Biologics, Inc., Silver Spring, MD), 219 with a non-biologic envelope (Tyrx®, Medtronic Inc., Monmouth Junction, NJ), and 71 with no envelope.

Results

Most patients had two or more infection risk factors (77.9% with any envelope vs. 52.1% with no envelope; P < 0.001). Factors significantly associated with the use of an envelope included the history of heart failure, systemic anticoagulant use, the use of high-power or more complex devices, and reoperations. The overall rate of adverse events was 9.2% (n = 42). Rates of infection and hematoma were 1.8% and 2.6%, respectively. A decision tree is proposed that may aid clinical decision-making when considering CIED envelope usage.

Conclusions

There were no significant differences between groups in overall or individual adverse event rates. These data provide insight into real-world clinical decisions regarding the use of CIED envelopes and support the use of antibiotic-eluting CIED envelopes to limit infection risk in high-risk patients.

## Introduction

The use of cardiac implantable electronic devices (CIEDs) has increased in recent years, as the indications for these devices have expanded [1]. Due to the frequently associated comorbidities in this expanding patient population, the increase in CIED implantation has led to an even greater increase in complication rates, including infection [2-5]. In recent studies, reported rates of CIED infection range from 0.7% to 4.6% following de novo implantations and up to 7% following reoperations, with even higher rates seen among patients with greater numbers of infection risk factors [4-12].

Understanding the impact of risk factors for CIED complications is essential to real-world clinical decision-making. Patients with profiles that suggest increased risk for adverse outcomes may benefit from additional prophylactic measures, such as the use of antibacterial CIED envelopes and/or envelopes that support the development of healthy vascularized surgical pockets. Risk factors for CIED pocket infection identified by previous studies include comorbidities (e.g. heart failure and renal failure), certain medications (e.g. oral anticoagulants), device-related factors (e.g. high-power devices), and procedural factors (e.g. reoperation) [3,5-8,10,13-15]. While evidence consistently identifies these risk factors as significant predictors of infection and other adverse events, there remains a need to better understand the use of patient risk profiles to inform clinical decisions, including whether to use CIED envelopes and which envelope to use, in individual patients.

Antibacterial CIED envelopes have been clinically available for several years, and are often used to stabilize the device in the surgical pocket and mitigate infection risk. Implanted biologic and non-biologic biomaterials both interact with the body, yet intrinsic characteristics of the implant material(s) used may impact the host response. Two CIED envelopes are clinically available in the United States. The first is a biologic envelope (CanGaroo®, Aziyo Biologics, Inc., Silver Spring, MD), which is constructed from two 4-ply sheets of decellularized, non-crosslinked, lyophilized extracellular matrix (ECM) derived from porcine small intestinal submucosa [16]. Prior to implantation, the CanGaroo envelope is rehydrated in a sterile isotonic solution such as saline, to which physicians may choose to add antibiotic(s). The second is an absorbable synthetic substrate (non-biologic) mesh (Tyrx™, Medtronic Inc., Monmouth Junction, NJ) coated with a bioresorbable polyarylate polymer containing the drug substances rifampin and minocycline [16]. Both the biologic and non-biologic envelopes have displayed similar antibiotic elution kinetic profiles in separate studies, and provide clinically meaningful levels of antibiotics to the surgical pocket following implantation [17-20]. Antibiotic-eluting non-biologic CIED envelopes have been shown to reduce the incidence of major infections in controlled trials [20], and a number of real-world clinical reports have been published describing the use of antibiotic-eluting biologic CIED envelopes for infection prevention strategies [21-25].

This manuscript reports the results of a retrospective, post-market, real-world observational review of patients undergoing CIED implantation by a single physician, using either the CanGaroo or Tyrx envelope, or no envelope. The aim of this study was to compare factors in patient selection, clinical outcomes, and complications between treatment groups in an effort to improve future patient care. An interim analysis of these data was previously presented as a meeting abstract at the 2021 American Heart Association Scientific Sessions.

## Materials and methods

Research design

Retrospective data from a single surgeon’s clinical experience at Piedmont Athens Regional Hospital in Athens, GA were collected on CIED implantation patients treated between March 2017 and December 2019 (CARE Plus, NCT04351269). The goal was to evaluate risk profiles and clinical outcomes of patients who received one of two antibacterial CIED envelopes (CanGaroo or Tyrx) or no envelope at the time of their CIED implantation procedure. Qualifying patients who underwent CIED implantation during the study period were identified and their records reviewed.

The protocol and any amendments were reviewed and approved by Western IRB (WIRB) and granted a waiver of informed consent and the Health Insurance Portability and Accountability Act of 1996 (HIPAA) due to its retrospective nature. This work was conducted in accordance with the ethical principles in the Declaration of Helsinki and conducted according to the US and international standards of Good Clinical Practice in accordance with applicable federal regulations, International Council for Harmonisation guidelines, and institutional research policies and procedures.

Surgical technique

CIED implantation was performed according to standard techniques, and the technique did not differ between the three patient groups. Full capsulectomy was performed for all patients undergoing reoperation. Patient selection for receiving an envelope, and which envelope to use, was left to the discretion of the implanting physician. The appropriate CIED envelope size was selected by the implanting physician and based upon the size of the CIED being implanted. In all cases, bacitracin was used to irrigate the surgical pocket.

For patients who received a CIED envelope (either biologic or non-biologic), the CIEDs were connected to the leads and the leads were secured to the underlying tissue before the CIED was placed into the selected envelope and subsequently implanted. For patients receiving the biologic envelope, the CanGaroo envelope was hydrated in a sterile saline solution containing gentamicin and vancomycin for one to two minutes prior to implantation. The pre- and post-procedure medication regimens, as well as clinical treatment, were performed according to the routine practice of the implanting physician and were the same for each of the three groups.

Data collected

Patient, procedural, and follow-up data were collected on standardized case report forms by site clinical personnel and reviewed by the investigator or qualified monitors.

Data collected at baseline included limited demographic information, a listing of associated comorbidities, and tabulation of infection risk factors (oral systemic anticoagulants, chronic steroid use, renal insufficiency, diabetes, peripheral vascular disease, obesity, malnutrition, smoking status, congestive heart failure, malignancy, use of temporary pacing, prior device infection, pocket re-entry, and device replacement/revision). Infection risk factors significantly associated with increased risk for CIED-related infections were identified from the published literature and numerically counted for each subject to group subjects by lower infection risk (those having zero to one risk factor) or higher infection risk (those having two or more risk factors) [3,5]. Procedural data, including the type of CIED implanted and other details, were also collected for each patient. Data on complications were collected from the procedural visit, and all follow-up and unscheduled visits, up to 12 months post-operation.

Outcome measures

Collected clinical outcomes included the incidence of pocket infection, superficial cellulitis, superficial surgical site infection, hematoma, lead dislodgement, and other complications. Pocket infection was defined as an infection requiring surgical intervention (e.g. system removal and pocket revision) or treatment with long-term antibiotic therapy (if system removal was not possible) to manage one of the following: (1) superficial cellulitis in the region of the CIED pocket with wound dehiscence, erosion, or purulent drainage; (2) deep incisional or organ/space (pocket) surgical site infection; (3) persistent bacteremia; or (4) endocarditis. Superficial cellulitis was defined as an infection involving the skin with pain or tenderness, localized swelling, redness, or heat without purulent drainage. Superficial surgical site infections were those involving only the skin and subcutaneous tissue surrounding the incision and either purulent drainage or positive cultures from the superficial incision that responded to a course of antibiotics but did not require surgical intervention.

Safety outcomes were determined by analysis of all device-related adverse events. Device-related events were defined as clinical signs, symptoms, or conditions that were deemed by the investigator to be causally related to the implantation or the performance of the envelope.

Statistical analysis

Statistical comparisons were evaluated between CanGaroo, Tyrx, and no envelope groups, and between any envelope and no envelope groups. Continuous variables were assessed for normality. The cohort was then described using means with standard deviations for continuous variables and counts with percentages for categorical variables. Independent samples t-tests were used to compare mean differences between groups. Categorical variables were compared using Pearson's chi-square tests for comparisons with expected cell counts greater than or equal to five. Fisher’s exact tests were reported if greater than or equal to one expected cell count was less than five. P-values < 0.05 were considered statistically significant. SPSS version 26 (IBM Corp., Armonk, NY) was used for statistical analyses.

## Results

A total of 466 patient charts with at least 12 months of follow-up were reviewed, and 455 subjects were included in the analysis. Eleven subjects were excluded from the analysis for the following reasons: a pre-existing CanGaroo envelope was already in place from the index procedure (n = 1); a CanGaroo envelope and a Tyrx envelope were previously used in the same index procedure (n = 2); and lack of complete follow-up for 12 months (n = 8).

Background characteristics

Patient demographics and medical histories are listed in Table 1. Statistically significant differences (P < 0.05) were identified between groups with regard to age, BMI and BMI category, the incidence of congestive heart failure/heart failure, and incidence of systemic anticoagulant use. For patients receiving envelopes, CanGaroo patients were older on average (74.0 years) compared to the Tyrx group (70.3 years), while Tyrx patients had a higher mean BMI (30.6 kg/m^2^) compared to the CanGaroo group (28.6 kg/m^2^). The proportion of patients with normal BMI (18.5 to <25 kg/m^2^) was greater in the CanGaroo group than in the other groups; conversely, there was a higher proportion of patients with obesity (30 to <40 kg/m^2^) in the no envelope group. A diagnosis of heart failure was more frequently noted in patients who received envelopes (P < 0.001). Systemic anticoagulant use was also significantly more common in patients who received envelopes (P = 0.004).

**Table 1:** Patient demographics and clinical characteristics All values are reported as n (%) unless otherwise noted. ^† ^There were no patients who identified as Native Hawaiian or Pacific Islander or American Indian or Alaska Native. ^‡ ^Fisher’s exact test. Statistically significant values are in bold.

Characteristic	Total	CanGaroo	Tyrx	No envelope	P-value	Any envelope	No envelope	P-value
	N = 455	n = 165	n = 219	n = 71		n = 384	n = 71	
Age, years, mean ± SD	72.3 ± 13.2	74.0 ± 13.2	70.3 ± 13.5	74.2 ± 11.0	0.009	71.9 ± 13.5	74.2 ± 11.0	0.124
Gender, male	288 (63.3)	101 (61.2)	140 (63.9)	47 (66.2)	0.740	241 (62.8)	47 (66.2)	0.581
BMI, kg/m^2^, mean ± SD	29.9 ± 6.9	28.6 ± 6.5	30.6 ± 7.2	31.0 ± 6.3	0.005	29.7 ± 7.0	31.0 ± 6.3	0.135
BMI category (kg/m^2^)					0.007			0.078
Underweight (<18.5)	5 (1.1)	2 (1.2)	2 (0.9)	1 (1.4)	-	4 (1.0)	1 (1.4)	-
Normal (18.5 to <25.0)	108 (23.7)	57 (34.5)	43 (19.6)	8 (11.3)	-	100 (26.0)	8 (11.3)	-
Overweight (25.0 to <30.0)	135 (29.7)	40 (24.2)	72 (32.9)	23 (32.4)	-	112 (29.2)	23 (32.4)	-
Obese (30.0 to <40.0)	170 (37.4)	55 (33.3)	81 (37.0)	34 (47.9)	-	136 (35.4)	34 (47.9)	-
Morbidly obese (40.0+)	37 (8.1)	11 (6.7)	21 (9.6)	5 (7.0)	-	32 (8.3)	5 (7.0)	-
Race^†^					0.510			0.531
White	390 (85.7)	143 (86.7)	183 (83.6)	64 (90.1)	-	326 (84.9)	64 (90.1)	-
Black or African American	39 (8.6)	16 (9.7)	20 (9.1)	3 (4.2)	-	36 (9.4)	3 (4.2)	-
Other or unknown	26 (5.7)	6 (3.6)	16 (7.3)	4 (5.6)	-	22 (5.7)	4 (5.6)	-
Ethnicity					0.480			0.756
Non-Hispanic or Latino	433 (95.2)	159 (96.4)	206 (94.1)	68 (95.8)	-	365 (95.1)	68 (95.8)	-
Hispanic or Latino	3 (0.7)	0 (0.0)	3 (1.4)	0 (0.0)	-	3 (0.8)	0 (0.0)	-
Unknown	19 (4.2)	6 (3.6)	10 (4.6)	3 (4.2)	-	16 (4.2)	3 (4.2)	-
Relevant medical history								
None	3 (0.7)	0 (0.0)	3 (1.4)	0 (0.0)	0.196	3 (0.8)	0 (0.0)	1.000^‡^
Hypertension	351 (77.1)	131 (79.4)	162 (74.0)	58 (81.7)	0.279	293 (76.3)	58 (81.7)	0.359
Obesity (BMI > 30 kg/m^2^)	207 (45.5)	66 (40.0)	102 (46.6)	39 (54.9)	0.097	168 (43.8)	39 (54.9)	0.092
Coronary artery disease	202 (44.4)	73 (44.2)	101 (46.1)	28 (39.4)	0.615	174 (45.3)	28 (39.4)	0.360
Heart failure	229 (50.3)	83 (50.3)	124 (56.6)	22 (31.0)	<0.001	207 (53.9)	22 (31.0)	<0.001
Systemic anticoagulant use	185 (40.7)	74 (44.8)	93 (42.5)	18 (25.4)	0.015	167 (43.5)	18 (25.4)	0.004
Diabetes	143 (31.4)	47 (28.5)	79 (36.1)	17 (23.9)	0.095	126 (32.8)	17 (23.9)	0.139
Renal insufficiency not requiring dialysis	77 (16.9)	29 (17.6)	38 (17.4)	10 (14.1)	0.784	67 (17.4)	10 (14.1)	0.487
Renal failure requiring dialysis	12 (2.6)	2 (1.2)	9 (4.1)	1 (1.4)	0.168	11 (2.9)	1 (1.4)	0.701^‡^
Current smoker	58 (12.7)	19 (11.5)	31 (14.2)	8 (11.3)	0.685	50 (13.0)	8 (11.3)	0.684
Chronic obstructive pulmonary disease	41 (9.0)	12 (7.3)	24 (11.0)	5 (7.0)	0.376	36 (9.4)	5 (7.0)	0.528
Peripheral vascular disease	28 (6.2)	9 (5.5)	16 (7.3)	3 (4.2)	0.577	25 (6.5)	3 (4.2)	0.597^‡^
Chronic steroid use	6 (1.3)	2 (1.2)	4 (1.8)	0 (0.0)	0.497	6 (1.6)	0 (0.0)	0.596^‡^
Prior device implant history								
Pre-procedure temporary pacing	4 (0.9)	1 (0.6)	3 (1.4)	0 (0.0)	0.503	4 (1.0)	0 (0.0)	0.388
Prior device infection (>12 months prior)	1 (0.2)	1 (0.6)	0 (0.0)	0 (0.0)	0.414	1 (0.3)	0 (0.0)	0.667
Presence of epicardial leads	2 (0.4)	0 (0.0)	2 (0.9)	0 (0.0)	0.339	2 (0.5)	0 (0.0)	0.542

Procedure- and device-related factors

Patients were treated using a wide range of procedures and devices (Table 2). There were 291 (64%) de novo implantations and 164 (36%) reoperations in this dataset. The rate of patients who underwent reoperative procedures differed significantly between groups: 89 (53.9%) patients in the CanGaroo group were undergoing reoperation versus 74 (33.8%) patients in the Tyrx group and one (1.4%) in the no envelope group (P < 0.001). De novo procedures were correspondingly more common in the Tyrx and no envelope groups. The majority of patients (n = 407, 89.5%) underwent CIED implantation alone; seven (1.5%) underwent pocket revision alone; one (0.2%) underwent device relocation; 34 (7.5%) had a lead addition, revision, or replacement; five (1.1%) underwent lead addition, revision, and/or replacement; and one patient (0.2%) had an unspecified procedure. Hemostatic agents were used in nearly all cases (n = 445, 97.8%), with no difference between groups.

**Table 2:** Surgical and device-related details Values are reported as n (%) unless otherwise noted. ^† ^No patients received subcutaneous implantable cardiac defibrillators (S-ICD) in this study. ^‡ ^Fisher’s exact test. Statistically significant values are in bold. ICD, implantable cardiac defibrillator; CRT-P, cardiac resynchronization therapy with pacemaker; CRT-D, cardiac resynchronization therapy with defibrillation.

Characteristic	Total	CanGaroo	Tyrx	No envelope	P-value	Any envelope	No envelope	P-value
	N = 455	n = 165	n = 219	n = 71		n = 384	n = 71	
High- vs. low-power CIED (n = 455)					<0.001			0.004
Low power	294 (64.6)	112 (67.9)	124 (56.6)	58 (81.7)	-	236 (61.5)	58 (81.7)	-
High power	156 (34.3)	50 (30.3)	93 (42.5)	13 (18.3)	-	143 (37.2)	13 (18.3)	-
Pocket/lead revision and/or lead replacement only	5 (1.1)	3 (1.8)	2 (0.9)	0 (0.0)	-	5 (1.3)	0 (0.0)	-
Low power (n = 294)					0.016			0.004
Pacemaker	264 (89.8)	97 (86.6)	109 (87.9)	58 (100.0)	-	206 (87.3)	58 (100.0)	
CRT-P	30 (10.2)	15 (13.4)	15 (12.1)	0 (0.0)	-	30 (12.7)	0 (0.0)	-
High power (n = 156)^†^					0.034			0.032^‡^
ICD	99 (63.5)	34 (68.0)	53 (57.0)	12 (92.3)	-	87 (60.8)	12 (92.3)	-
CRT-D	57 (36.5)	16 (32.0)	40 (43.0)	1 (7.7)	-	56 (39.2)	1 (7.7)	-
De novo procedure	291 (64.0)	76 (46.1)	145 (66.2)	70 (98.6)	<0.001	221 (57.6)	70 (98.6)	<0.001
Reoperative procedure	164 (36.0)	89 (53.9)	74 (33.8)	1 (1.4)		163 (42.4)	1 (1.4)	
CIED procedure type								
De novo procedure (n = 291)					0.024			0.040
Low power	209 (71.8)	58 (76.3)	94 (64.8)	57 (81.4)	-	152 (68.8)	57 (81.4)	-
High power	82 (28.2)	18 (23.7)	51 (35.2)	13 (18.6)	-	69 (31.2)	13 (18.6)	-
Reoperative procedure (n = 164)					0.091			0.627
Low power	85 (51.8)	54 (60.7)	30 (40.5)	1 (100.0)	-	84 (51.5)	1 (100.0)	-
High power	74 (45.1)	32 (36.0)	42 (56.8)	0 (0.0)	-	74 (45.4)	0 (0.0)	-
Pocket/lead revision and/or lead replacement only	5 (3.0)	3 (3.4)	2 (2.7)	0 (0.0)	-	5 (3.1)	0 (0.0)	-

Significant differences were identified between groups with regard to the type of device and procedure. Overall, antibacterial envelopes were used more often when high-power (P = 0.004) or more complex devices were used (cardiac resynchronization therapy with pacemaker (CRT-P) vs. pacemaker, P = 0.004; cardiac resynchronization therapy with defibrillation (CRT-D) vs. implantable cardiac defibrillator (ICD), P = 0.032). In de novo procedures, envelopes were used more often in patients receiving high-power devices (P = 0.040).

Infection and other adverse events

A total of 42 (9.2%) adverse events were identified during the follow-up period: 17 (10.3%) in the CanGaroo group, 23 (10.5%) in the Tyrx group, and two (2.8%) in the no envelope group (Table 3). No adverse events were reported after 252 days of follow-up. There were two major infections (0.4%), both in the CanGaroo group: one on postoperative day (POD) 10 following a reoperative procedure in a high-risk patient, and one at POD 65. There were six minor CIED infections (1.3%): two in the CanGaroo group (1.2%) and four in the Tyrx group (1.8%). All minor infections occurred within POD two to four, except for one at POD 68. In each envelope group, one of the minor infections occurred in patients undergoing reoperation. However, there were no significant differences between groups in the incidence of infection.

**Table 3:** Adverse events All values are reported as n (%) unless otherwise noted. ^† ^Superficial surgical site infection. ^‡ ^Fisher’s exact test. ^§ ^All hematoma occurring in the study population required intervention. Statistically significant values are in bold. CIED, cardiac implantable electronic device.

Adverse event	Total	CanGaroo	Tyrx	No envelope	P-value	Any envelope	No envelope	P-value
	N = 455	n = 165	n = 219	n = 71		n = 384	n = 71	
Infection, total	8 (1.8)	4 (2.4)	4 (1.8)	0 (0.0)	-	8 (2.1)	0 (0.0)	-
Major CIED infection	2 (0.4)	2 (1.2)	0 (0.0)	0 (0.0)	0.171	2 (0.5)	0 (0.0)	1.000^‡^
Pocket infection	2 (0.4)	2 (1.2)	0 (0.0)	0 (0.0)	-	2 (0.5)	0 (0.0)	-
Minor CIED infection^†^	6 (1.3)	2 (1.2)	4 (1.8)	0 (0.0)	0.497	6 (1.6)	0 (0.0)	0.596^‡^
Hematoma^§^	12 (2.6)	8 (4.8)	4 (1.8)	0 (0.0)	0.060	12 (3.1)	0 (0.0)	0.228
Other adverse events (total)	22 (4.8)	5 (3.0)	15 (6.8)	2 (2.8)	0.155	20 (5.2)	2 (2.8)	0.552^‡^
Lead dislodgement	5 (1.1)	1 (0.6)	3 (1.4)	1 (1.4)	0.749	4 (1.0)	1 (1.4)	0.574^‡^
Lead revision	1 (0.2)	0 (0.0)	1 (0.5)	0 (0.0)	0.583	1 (0.3)	0 (0.0)	1.000^‡^
Erythema/fever	1 (0.2)	0 (0.0)	0 (0.0)	1 (1.4)	0.067	0 (0.0)	1 (1.4)	0.156^‡^
Hemothorax	1 (0.2)	0 (0.0)	1 (0.5)	0 (0.0)	0.583	1 (0.3)	0 (0.0)	1.000^‡^
Site drainage	1 (0.2)	1 (0.6)	0 (0.0)	0 (0.0)	0.414	1 (0.3)	0 (0.0)	1.000^‡^
Lead perforation	2 (0.4)	2 (1.2)	0 (0.0)	0 (0.0)	0.171	2 (0.5)	0 (0.0)	1.000^‡^
Thrombosis	1 (0.2)	1 (0.6)	0 (0.0)	0 (0.0)	0.414	1 (0.3)	0 (0.0)	1.000^‡^
All-cause death	10 (2.2)	0 (0.0)	10 (4.6)	0 (0.0)	0.004	10 (2.6)	0 (0.0)	0.374

A total of 12 (2.6%) hematoma were reported, all of which required intervention. All hematoma (100%) occurred in patients undergoing reoperation, and most (n = 8, 66.7%) of these patients received high-power devices and were taking systemic anticoagulants (n = 11, 91.7%). The occurrence of hematoma was seen only in the envelope groups (CanGaroo: n = 8, 4.8%; Tyrx: n = 4, 1.8%), but the numerical difference between envelope groups was not statistically significant.

When subjects were categorized by their number of infection risk factors, significant differences were noted between treatment groups (Table 4). Higher proportions of patients in both envelope groups had two or more infection risk factors (envelope: 77.9% vs. no envelope: 52.1%; P < 0.001), whereas a higher proportion of patients in the no envelope group had zero to one risk factor (envelope: 22.1% vs. no envelope: 47.9%; P < 0.001). The mean number of infection risk factors for the CanGaroo and Tyrx envelope groups was identical (2.9 ± 1.7).

**Table 4:** Analysis by number of infection risk factors Statistically significant values are in bold.

Number of infection risk factors	Total	CanGaroo	Tyrx	No envelope	P-value	Any envelope	No envelope	P-value
	N = 455	n = 165	n = 219	n = 71		n = 384	n = 71	
Mean ± SD	2.7 ± 1.7	2.9 ± 1.7	2.9 ± 1.7	1.9 ± 1.4	<0.001	2.9 ± 1.7	1.9 ± 1.4	<0.001
0-1, n (%)	119 (26.2%)	39 (23.6%)	46 (21.0%)	34 (47.9%)	<0.001	85 (22.1%)	34 (47.9%)	<0.001
≥2, n (%)	336 (73.8%)	126 (76.4%)	173 (79.0%)	37 (52.1%)		299 (77.9%)	37 (52.1%)	

There were no instances of device migration or erosion, Twiddler’s syndrome, or need for pocket revision following implantation in any of the groups. The only significant difference between groups in adverse event rates was all-cause deaths, all 10 of which occurred in the Tyrx group (4.6%, P = 0.004). These deaths were not considered to be related to the device or CIED procedure.

## Discussion

Biologic and non-biologic CIED envelopes have been widely used in the clinical setting for several years and continue to gain popularity among implanting physicians. However, best practices regarding when to use an envelope and which envelope to consider for individual patients remain to be fully elucidated. This study examined factors underlying the decision to use an antibacterial CIED envelope, which envelope to use (biologic or non-biologic), and the 12-month real-world outcomes of a large patient sample undergoing CIED implantation by a single physician.

Overall, the management patterns revealed in this analysis indicate a preference for use of an envelope in patients with more infection risk factors and/or who are managed with high-power or more complex devices. Indeed, patients with established infection risk factors such as heart failure or the use of systemic anticoagulants were significantly more likely to receive a CIED envelope (P < 0.001 and P = 0.004, respectively). When per-patient infection risk factors were summed, patients with two or more infection risk factors were significantly more likely to receive a CIED envelope compared to those with zero to one risk factor (P < 0.001), and the mean number of risk factors was significantly greater in the any envelope group compared to the no envelope group (mean 2.9 vs. 1.9; P < 0.001). Similarly, the use of high-power devices was significantly more common in the any envelope compared to the no envelope group (P = 0.004), as was the use of more complex devices, such as CRT-P versus pacemakers among low-power devices (P = 0.004) and CRT-D versus ICD among high-power devices (P = 0.032). These treatment patterns may reflect the relative benefits of the different envelopes as perceived by the implanting physician based on patient and procedural factors.

Complication rates with CIED envelopes

The adverse event rate in the reviewed patient population was relatively low (n = 42, 9.2%), with no significant differences between groups (CanGaroo: n = 17, 10.3%; Tyrx: n = 23; 10.5%; no envelope: n = 2, 2.8%). These rates align with those reported in the literature, which for major adverse events (i.e. those requiring intervention), range from 1.6% to 15.3% [4-12].

Individual adverse events, including hematoma and infection (see below section), also did not differ significantly between groups. The incidence of hematoma (n = 12, 2.6%), all of which occurred between both envelope groups, was consistent with previous reports from major studies. For example, a recent analysis of the World-wide Randomized Antibiotic Envelope Infection Prevention Trial (WRAP-IT) reported a 2.2% incidence of hematoma in the combined study population (envelope and no envelope, N = 6800) [26]. This analysis also demonstrated a significant relationship between hematoma and increased risk for infection in the control (i.e. no envelope) group, and among patients with hematoma, a significantly lower risk for major infection in the envelope group compared to the control group (2.5% vs. 13.1%; P = 0.030). The fact that no hematoma led to infection in either of the envelope groups in our reported experience aligns with the findings of the WRAP-IT study and supports the beneficial role of antibacterial envelopes in reducing the risk of infectious consequences of hematoma.

CIED infection

CIED infections are a serious complication that can result in significant morbidity and mortality, with corresponding increases in healthcare costs [1,11,27,28]. One analysis of the National Inpatient Sample database identified a mortality rate of 4.5% when lead extraction was required; the mortality rate was the greatest in patients over 85 years of age (5.3%), compared to patients aged 18-44 years (2.5%; P < 0.001) [29]. Between 2003 and 2011, that analysis documented a significant increase in the number of hospitalizations for CIED infections and a 53% increase in mean charges for infection-related hospitalizations. Overall, the costs of managing CIED infections have been estimated to range from US$22,856 to US$77,397 per patient, with average adjusted annual medical costs 2.4-times greater for patients with a CIED infection [27,28]. Antibacterial envelopes are designed to reduce infection risk and mitigate these serious consequences.

The incidence of all infections in our total dataset (1.8%) is consistent with rates previously reported in the literature, which range from 0.7% to 4.6% following de novo implantation, and as high as 7% following reoperation [4-12]. Prior studies have demonstrated that CIED infection rates vary based on procedure- and patient-related factors, such as de novo placements versus reoperations, the type of device implanted, patient comorbidities, and the use of perioperative antibiotics and/or antibiotic-eluting envelopes [8]. Despite the low infection rate, our patient population had a relatively high infection risk: 36.0% of the procedures performed were reoperations and 73.8% of all patients had two or more infection risk factors. Our findings are also consistent with major prospective studies, such as WRAP-IT, which compared the use of non-biologic CIED envelopes to no envelope, and Prevention of Arrhythmia Device Infection Trial (PADIT), which compared different approaches to infection prophylaxis, without the use of CIED envelopes [20,30]. Both of these studies enrolled patients with high infection risk and reported an approximately 1% rate of major infections, comparable to our results (0.4% major infections), and suggested a baseline for infection risk in the context of aggressive infection prophylaxis. As noted, no adverse events (including infection) were reported after 252 days of follow-up in our dataset; this finding is consistent with outcomes of the WRAP-IT study, which found continued suppression of infection risk through long-term follow-up in the envelope group [20].

The fact that no infections occurred in the no envelope group may reflect that the implanting physician used effective prophylactic techniques and appropriate antibacterial envelope patient selection based on the presence of infection risk factors, as indicated by the fact that 89% of patients with two or more infection risk factors received a CIED envelope. The lack of statistical differences between groups in infection rate may also reflect the efficacy of total infection-prevention measures and suggests that biologic and non-biologic antibacterial envelopes are similarly effective in reducing infections in high-risk patients.

ECM biomaterials, infection risk, and pocket vascularization

Although the biologic and non-biologic envelopes used separately in this experience demonstrated similar adverse event rates, differences in the materials from which these envelopes are constructed may have important implications for the host response to the material and therefore potentially patient selection.

In pre-clinical and clinical studies, non-biologic (absorbable and non-absorbable) surgical materials have been shown to trigger a robust foreign body response, including chronic inflammation, which leads to encapsulation of the material in fibrotic, hypovascularized tissue [31-35]. In fact, the first commercially available CIED envelope, the Parsonnet pouch (Bard Peripheral Vascular Inc., Tempe, AZ), was specifically designed to exploit the foreign body response to create a robust fibrous capsule to secure the device [35].

Conversely, biologic materials (such as those made from non-crosslinked ECM) have very different effects after implantation. Non-crosslinked ECM-based biomaterials have been shown to foster tissue integration and vascular ingrowth, a modulated inflammatory response, and rapid clearance of bacteria, thereby contributing to the development of well-vascularized tissue and attenuated infection risk [32,36-39]. The mechanisms underlying these effects include the release of bioavailable growth factors and antimicrobial elements from the ECM during remodeling [37-40]. Among other actions, these growth factors stimulate angiogenesis, which allows host immune cells to access the area undergoing remodeling [36,40,41]. The immunomodulatory process that occurs during ECM remodeling fosters the development of highly vascularized tissue, which in the case of a CIED pocket, can support long-term pocket health [32,36,40]. Whereas the immune response to non-biologic surgical materials is characterized by a predominantly M1 macrophage phenotype that promotes inflammation, non-crosslinked ECM biologic biomaterials elicit a predominantly M2 macrophage phenotype, which is anti-inflammatory and promotes immunoregulation and constructive remodeling of tissues [36,42-44]. Thus, the CanGaroo ECM envelope was designed to stimulate healthy, vascularized tissue ingrowth to stabilize the device within the pocket, which may limit the risk for device migration or erosion, and possibly facilitate device removal during the future exchange or revision procedures due to attenuated foreign body response [16,21].

For these reasons, physicians may prefer ECM-based materials for patients and indications with a higher risk for adverse outcomes such as infection or erosion, or for patients who may benefit from having a layer of soft tissue surrounding their device. Accordingly, a recent study of patient characteristics associated with the use of the CanGaroo envelope identified a physician preference for these devices in patients who were elderly with poor tissue quality, had a history of prior device infection, or had multiple infection risk factors [21].

The inclusion of antibiotics in CIED envelopes is a newer innovation that has the advantage of providing short-term, local antibiotic elution where it is most needed. In preclinical studies, biologic ECM envelopes hydrated in antibiotic solutions showed a biphasic pattern of antibiotic release, with an initial bolus followed by sustained release over several days [17,18]. Because many CIED infections likely begin at the time of implantation, high and sustained local antibiotic concentrations in the pocket may be optimal to prevent infection. Both types of envelopes used in this physician’s practice (biologic and non-biologic) have been shown to elute antibiotics with similar elution kinetics in separate studies [17-20]. The non-biologic envelopes are already impregnated with rifampicin and minocycline, whereas the biologic envelopes were hydrated in a saline solution containing gentamicin and vancomycin prior to implantation. One potential advantage of biologic envelopes is that the implanting physician can select specific antibiotics based on patient or local factors, whereas the non-biologic envelope is limited to the agents impregnated during manufacture.

Clinical decision-making: use of an antibacterial CIED envelope vs. no envelope

**Figure 1:**
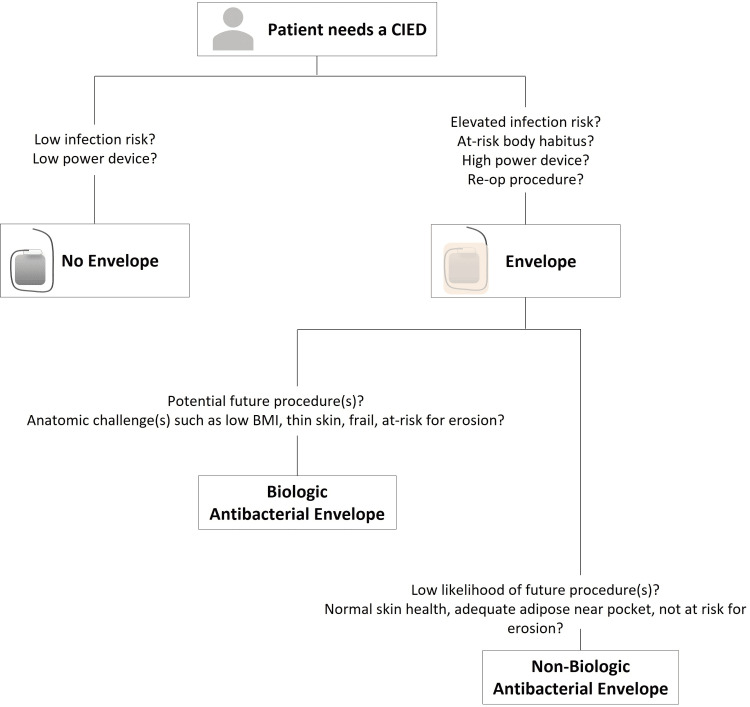
Patient selection decision tree for CIED envelope use Proposed envelope selection according to the patient and procedure-specific factors listed and based on use patterns in this clinical experience in combination with the published literature. Patients with low infection risk or receiving a low-power device generally do not receive an envelope. Patients receiving de novo or reoperative procedures who are at risk for infection or have at-risk body habitus and who are either receiving a high-power device, reoperative procedure, or a de novo device with expected future operations generally receive an envelope. Patients who receive envelopes either receive a biologic or non-biologic antibacterial envelope. Biologic antibacterial envelopes are generally used for patients who are expected to have a future procedure(s), or for patients with anatomic challenges such as low BMI, thin skin, frail, or at risk for device erosion. Non-biologic antibacterial envelopes are generally used in patients who have a low likelihood of a future procedure(s) and have normal skin health, adequate adipose present near the pocket, and who are not at risk for device erosion. CIED, cardiac implantable electronic device.

Taken together, the results of this analysis indicate that antibiotic-eluting CIED envelopes are preferred for patients and devices with a higher risk for infection, as also demonstrated by previous studies [13,16,20-24]. To aid clinical decision-making and potentially improve patient care, we combined our practice patterns, the results from this analysis, and results from previous studies to suggest a decision tree for CIED antibacterial envelope usage, as illustrated in Figure 1 [3,5,11,12,16,20-22,26,45-50]. The collective current and previous bodies of evidence support the flow of this decision tree; however, it is by no means a complete work; future studies are still needed to fully appreciate all patient and procedural factors that should be considered for the implantation of these antibacterial envelopes. In this scheme, patients with lower infection risk and who are managed with low-power devices may be considered for CIED implantation without an envelope. Use of a CIED envelope may be considered for patients with elevated infection risk, at-risk body habitus, those receiving high-power devices, and/or those undergoing reoperative procedures.

Clinical decision-making: use of biologic vs. non-biologic antibacterial CIED envelopes

Our current clinical experience and others have found that the use of biologic or non-biologic antibacterial envelopes results in similar clinical outcomes. Additional data are still needed to determine patient and procedural factors that benefit the most from each envelope. However, factors that this implanting physician considers a biologic envelope beneficial include patients with the potential for future procedures (such as patients who may need a device changeout later in life), or the presence of risk factors for device erosion or device-related discomfort (such as low BMI, thin skin, or frail patients). In these patients, a healthy, well-vascularized tissue layer in the device pocket may be advantageous. This physician normally considers non-biologic envelopes for patients who may benefit from an antibiotic-eluting envelope but are not as likely to undergo future procedures and are not at risk for device erosion. As noted previously, non-biologic surgical materials can be associated with a robust foreign body response and the formation of a fibrotic capsule, which may complicate future reoperations and typically require capsulectomy, possibly compromising tissue quality around the implant. In contrast, non-crosslinked ECM-based biologic materials have been shown to promote angiogenesis and an immunomodulatory response associated with constructive remodeling. The formation of a more cellularized and vascularized pocket may facilitate future procedures and reduce the risk of device erosion or reoperative infection [15,28,40,48-50].

Limitations

The major limitations of this retrospective clinical experience are its non-randomized design, relatively limited duration of follow-up, and single-physician design. The lack of randomization introduces bias in the selection of patients for implantation with CIED envelopes and in the choice between biologic and non-biologic envelopes. Due to these limitations, the intent of this analysis was to describe physician practice patterns, rather than demonstrate the superiority of one approach over another. Further studies are still needed to fully explore clinical decision-making considerations for envelope usage, thus our proposed decision tree is solely suggested as a starting point based on our current practices and the currently available data. Finally, the duration of follow-up may not have captured late adverse events, limiting data on longer-term outcomes.

## Conclusions

This analysis provides insight into real-world clinical decision-making regarding the use of antibacterial CIED envelopes. It also provides further evidence that antibiotic-eluting CIED envelopes can mitigate infection risk, as even in a high-risk population, the use of biologic and non-biologic antibiotic-eluting envelopes produced a low rate of major infections (<1%). These findings illustrate the decision-making process for determining usage of a CIED envelope and employment of a biologic or non-biologic envelope, based on patient- and procedure-related factors in our practice and the available related literature. Additional studies will further clarify factors that will optimize the costs, benefits, and appropriate use of different antibiotic-eluting CIED envelopes in specific clinical scenarios.
